# The changes of inflammatory mediators and vasoactive substances in dairy cows’ plasma with pasture-associated laminitis

**DOI:** 10.1186/s12917-020-02319-1

**Published:** 2020-04-23

**Authors:** Xianhao Zhang, Jiafeng Ding, Yuepeng Li, Qiaozhi Song, Shuaichen Li, Muhammad Abid Hayat, Jiantao Zhang, Hongbin Wang

**Affiliations:** Heilongjiang Key Laboratory for Laboratory Animals and Comparative Medicine, Department of Veterinary Surgery, College of Veterinary Medicine, Northeast Agricultural University, Harbin, P. R. China

**Keywords:** Dairy cow, Laminitis, Inflammatory mediator, Vasoactive substance, Elisa

## Abstract

**Background:**

Hoof disease is one of the three major diseases that often occur in dairy cows. The impact of this disease on dairy farming is second only to mastitis. Laminitis is a diffuse, aseptic, serous, non-purulent inflammation of the dermal papillae and vascular layers of the cow’s hoof wall. In the pasture, laminitis occurs mostly in the laminae, that is, inside the hoof shell. No lesions can be seen on the surface. Therefore, laminitis cannot attract the attention of veterinarians. However, laminitis has become a major factor that seriously affects the health and welfare of dairy cows, making it an important cause of hindering the performance of dairy cows.

**Methods:**

The study was conducted at a dairy farm in Harbin, Heilongjiang province, China. We selected a sample of the laminitis cows based on the veterinary diagnosis, took blood from the jugular vein and then separated the plasma, and measured the index with the Elisa kit. In this study, the markers of inflammatory and vasoactive substances status in dairy cows consisted of subclinical laminitis (SCL, *n* = 20), chronic laminitis (CL, *n* = 20) and healthy dairy cows (CON, *n* = 20) under the local management conditions were investigated.

**Results:**

Compared with healthy cattle, HIS, IL-6, LPS, and TNF-α in subclinical laminitis group significantly increased (*P* < 0.05), especially HIS, LPS, TNF-α (*P* < 0.01); in chronic laminitis cows, COX-2, HIS, IL-6, LPS, and TNF-α increased significantly (*P* < 0.05), especially COX-2, HIS, TNF-α (*P* < 0.01). iNOS (*P* < 0.05), TXB2 (*P* < 0.01) in chronic laminitis cows had significantly increased.

**Conclusion:**

This study reported for the first time that pasture laminitis was divided into subclinical laminitis and clinical chronic laminitis. Through research on the inflammatory factors and vasoactive substances of dairy cows, it is found that there is a close relationship between them, which affects the metabolic cycle of dairy cows. These indicators are abnormally expressed and cause hoof microcirculation disorders.

## Background

Lameness is the main health problem in the dairy industry worldwide [1]. A crucial reason for lameness is subclinical laminitis (SCL), characterized by claw lesions such as hemorrhages of the sole and the white line, and sole ulcers [2]. Subclinical laminitis resulted in low milk production, poor health and reduced reproductive performance [3], which adversely affect the economic return of dairy cows. Another cause of lameness is the chronic laminitis, diagnosed by deformation of the hoof; the dorsal wall flattens and becomes concave along its length. Most of the abnormal shape is caused by the rotation and sinking of the pedal bone inside the claw that changes the growth direction. This deformation causes more pressure to be exerted on the posterior region of the sole.

Animals at the highest risk show a metabolic predisposition, including obesity and insulin resistance, related to that observed in humans [4]. Similar pathological mechanisms that cause cardiovascular disease in human metabolic syndrome, including changes in insulin signaling, inflammatory mediators, and vasoactive substances, could potentially contribute to laminitis. The inflammatory response will stimulate the pathological changes of blood vessels, which will change the fluidity of the blood, such as lamellae, reducing the use of nutrients by cells and signal transduction of cellular functions [5]. Reactive oxygen mediates inflammatory signals, while also weakening endothelial function and increasing tissue sensitivity to endotoxin or endotoxin-induced damage [5, 6].

In human diabetes and insulin resistance syndrome, inflammatory factors and vascular dysfunction are major pathological changes. The vasoconstriction, coagulant activity, and chronic vascular remodeling demonstrate vascular dysfunction. Insulin and obesity increase the secretion of inflammatory factors, affecting the production of nitric oxide (NO) which results in endothelial dysfunction [7]. The gluconeogenesis and sugar accumulation process of endothelial cells in diabetic patients can exacerbate vascular endothelial dysfunction (glucotoxicity). Glycotoxicity promotes the formation of advanced glycation end products (AGEs), further reducing NO concentration while promoting the production of reactive oxygen species and endothelial inflammatory factors. In animal and human experimental models, insulin resistance reduces capillary regenerative capacity and promotes vasoconstriction [7] Insulin resistance has a dual effect on blood vessels and inflammatory factors, and changes in either of them can cause laminitis by affecting blood circulation or inducing inflammatory factors and oxidative stress.

However, many previous studies have shown the overall situation of pasture-associated laminitis and no specific classification of laminitis conducted. In this study, we measured the changes of inflammation mediators and vasoactive substances in the plasma of the dairy cows with subclinical and chronic laminitis, aiming to discuss the correlations between selected parameters and pathogenesis of laminitis.

## Results

A comparison between the SCL, CL, and control groups was shown in Table 1 and Table 2. We examined a total of 60 cows, including subclinical laminitis, chronic laminitis and healthy without any symptoms with 20 animals in each group.

**Table 1:** Mean (SD) measurements of inflammatory agents in 20 dairy cows with subclinical laminitis, 20 dairy cows with chronic laminitis and 20 healthy dairy cows

Measurement	SCL	CL	CON
PGI-2 (pg/ml)	428.32 ± 123.11	238.37 ± 174.63	496.79 ± 168.10
COX-2 (ng/ml)	34.00 ± 9.13	63.91^**^ ± 22.34	37.44 ± 13.74
HIS (μg/ml)	4.96^**^ ± 1.19	5.47^**^ ± 1.03	3.97 ± 0.89
IL-1β (ng/ml)	69.05 ± 22.18	60.88 ± 12.66	67.48 ± 13.23
IL-6 (ng/ml)	30.56^*^ ± 6.30	34.60^*^ ± 5.99	25.76 ± 8.84
LPS (ng/ml)	815.07^**^ ± 107.75	749.97^*^ ± 192.76	684.23 ± 80.53
Tnf-α (ng/ml)	481.61^**^ ± 93.18	476.67^**^ ± 58.59	387.83 ± 63.15

The subclinical group (SCL) and the chronic group (CL) were compared with the healthy group (CON), and there was no comparison between the subclinical group and the chronic group. * indicates that the difference is significant (*P* < 0.05), and ** indicates that the difference is extremely significant (*P* < 0.01)

**Table 2:** Mean (SD) measurements of vasoactive substances in 20 dairy cows with subclinical laminitis, 20 dairy cows with chronic laminitis and 20 healthy dairy cows

Measurement	SCL	CL	CON
5-HT (ng/ml)	33.11 ± 17.33	21.85 ± 15.12	35.11 ± 16.63
ET-1 (pg/ml)	40.96 ± 18.07	38.91 ± 12.66	52.63 ± 15.04
iNOS (ng/ml)	5.86 ± 1.44	7.00^*^ ± 2.69	5.90 ± 3.10
TXB_2_ (pg/ml)	193.05 ± 34.84	291.80^**^ ± 50.68	189.47 ± 46.40

The subclinical group (SCL) and the chronic group (CL) were compared with the healthy group (CON), and there was no comparison between the subclinical group and the chronic group. * indicates that the difference is significant (*P* < 0.05), and ** indicates that the difference is extremely significant (*P* < 0.01)

### Inflammatory mediators

**Fig. 1:**
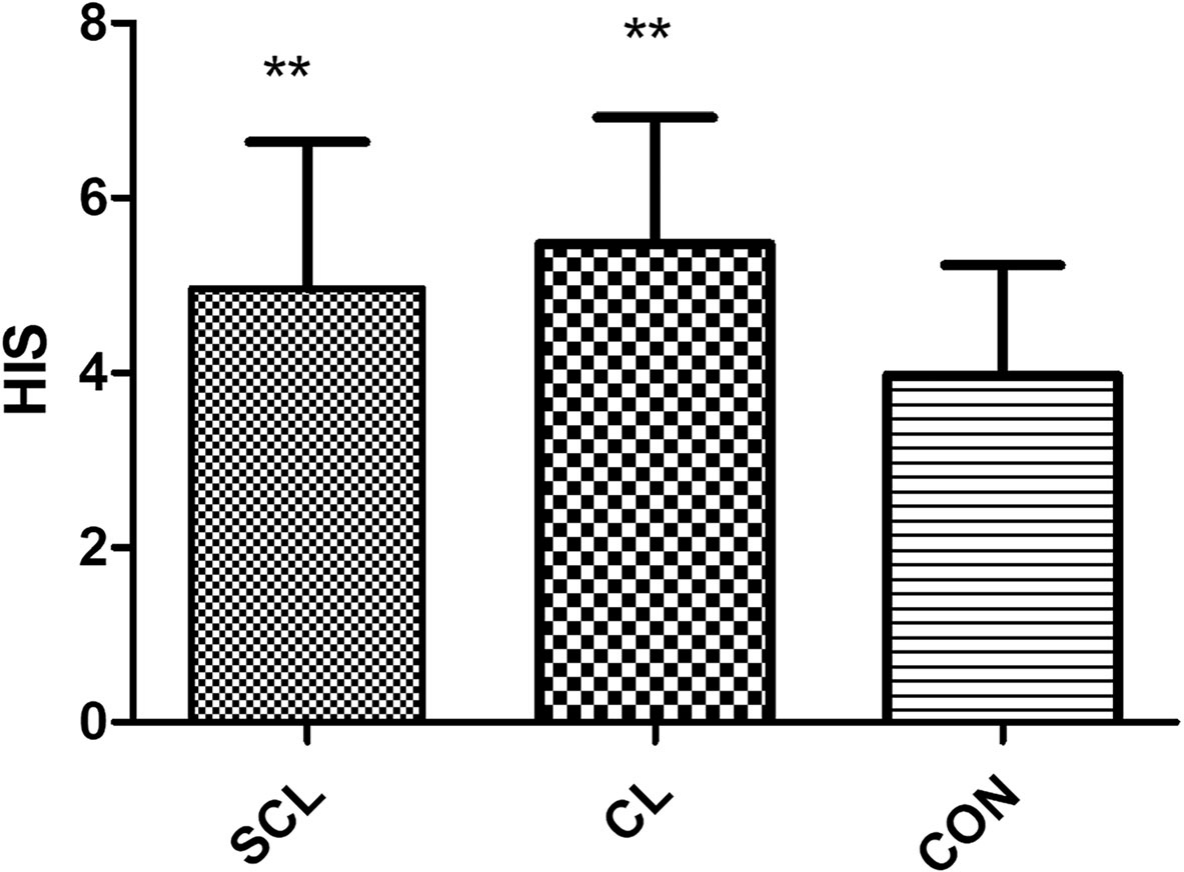
column bar plot comparing the distribution of: Histamine (HIS), in 20 dairy cows with subclinical laminitis, 20 dairy cows with chronic laminitis and 20 healthy dairy cows. The error bar shows with a line, symbol * indicate statistically significant differences between group

**Fig. 2:**
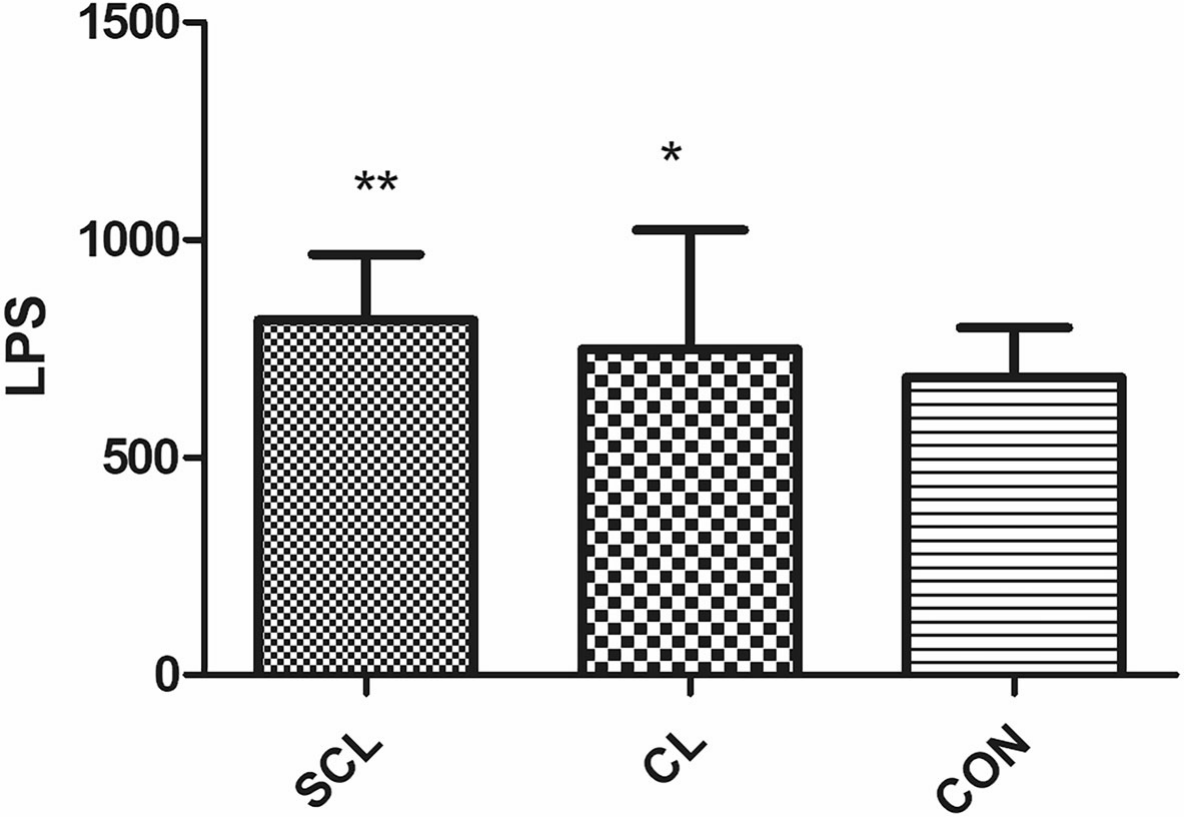
column bar plot comparing the distribution of: Lipopolysaccharide (LPS), in 20 dairy cows with subclinical laminitis, 20 dairy cows with chronic laminitis and 20 healthy dairy cows. The error bar shows with a line, symbol * indicate statistically significant differences between groups

**Fig. 3:**
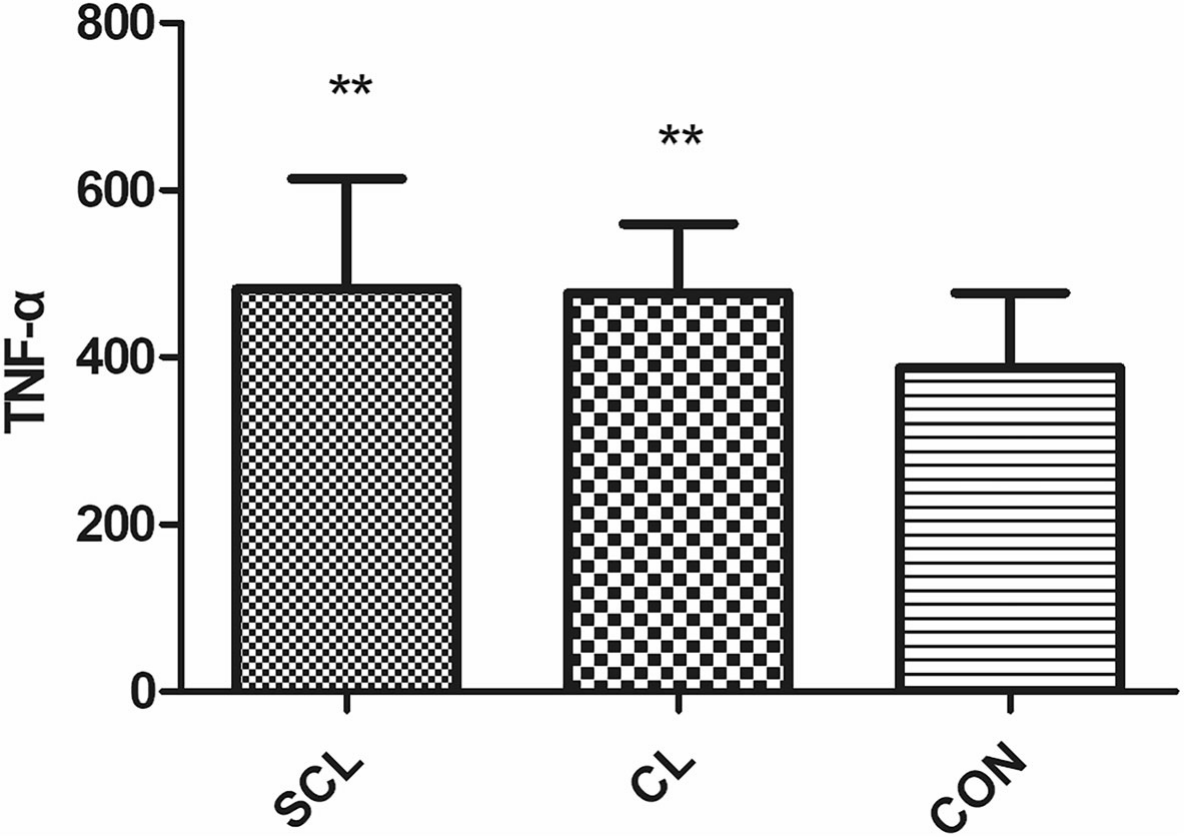
column bar plot comparing the distribution of: tumor necrosis factor-α (Tnf-α), in 20 dairy cows with subclinical laminitis, 20 dairy cows with chronic laminitis and 20 healthy dairy cows. The error bar shows with a line, symbol * indicate statistically significant differences between groups

**Fig. 4:**
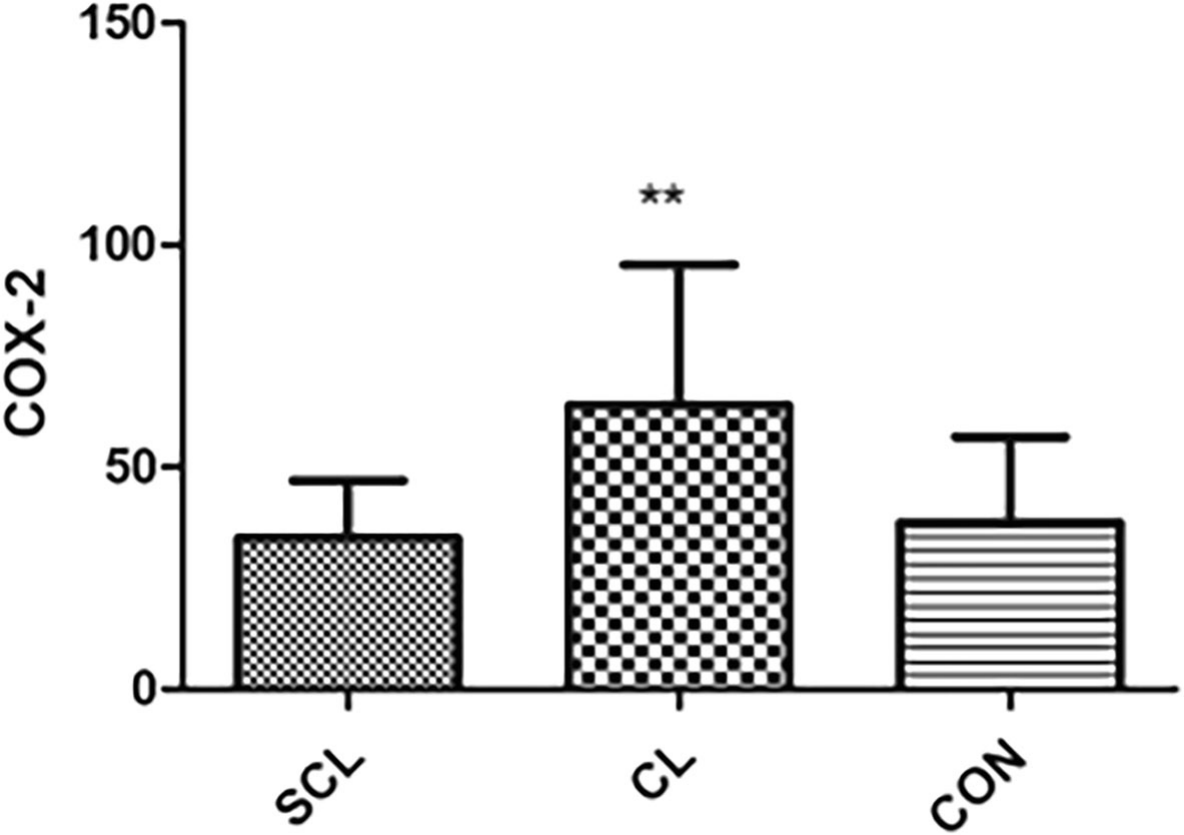
column bar plot comparing the distribution of: Cyclooxygenase-2 (COX-2), in 20 dairy cows with subclinical laminitis, 20 dairy cows with chronic laminitis and 20 healthy dairy cows. The error bar shows with a line, symbol * indicate statistically significant differences between groups

**Fig. 5:**
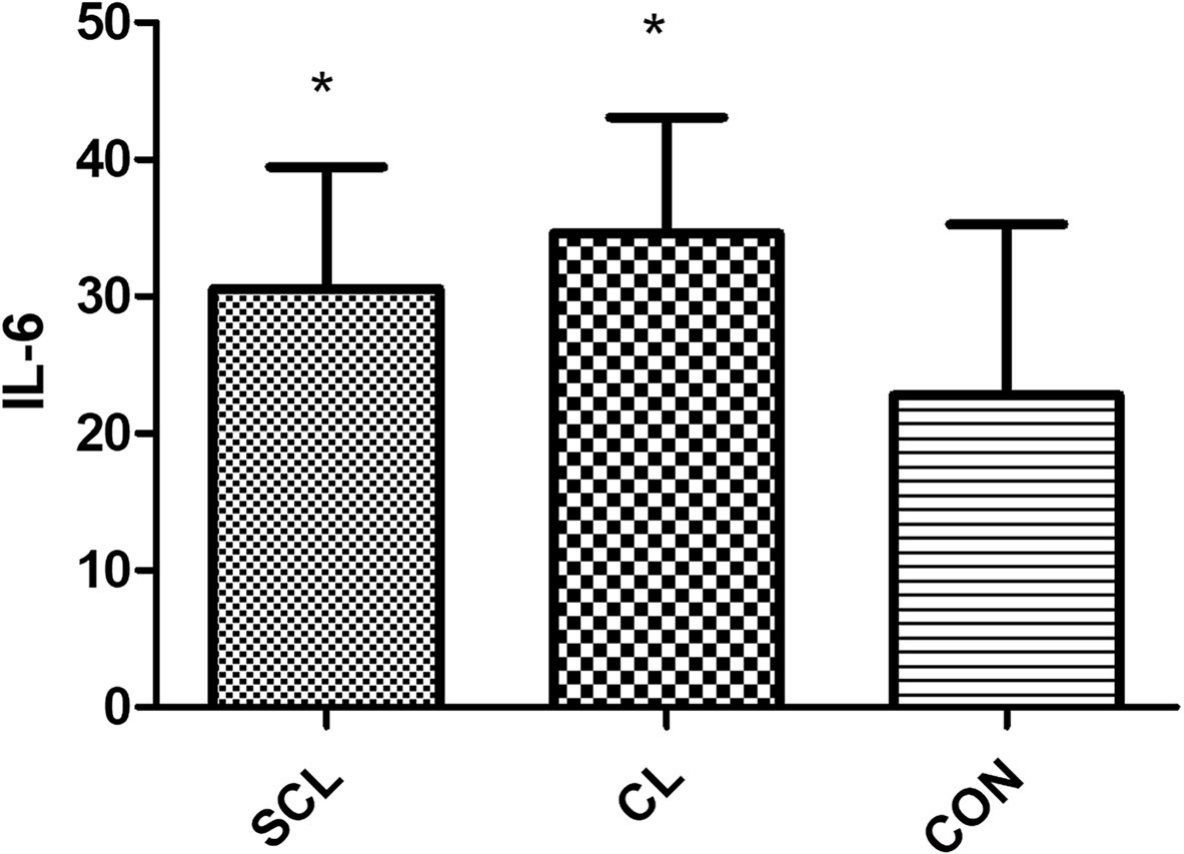
column bar plot comparing the distribution of: Interleukin-6 (IL-6), in 20 dairy cows with subclinical laminitis, 20 dairy cows with chronic laminitis and 20 healthy dairy cows. The error bar shows with a line, symbol * indicate statistically significant differences between groups

Compared with healthy cows, HIS, IL-6, LPS, and TNF-α in subclinical laminitis group significantly increased (*P* < 0.05), extremely significant increased HIS, LPS, TNF-α (*P* < 0.01) as shown in (Figs. 1, 2, 3, 4, 5); compared with healthy cows, COX-2, HIS, IL-6, LPS, and TNF-α in chronic laminitis cows were significantly increased (*P* < 0.05), extremely significant increased COX-2, HIS, TNF-α (*P* < 0.01) as shown in (Figs. 1, 2, 3, 4, 5).

### Vasoactive substances

**Fig. 6:**
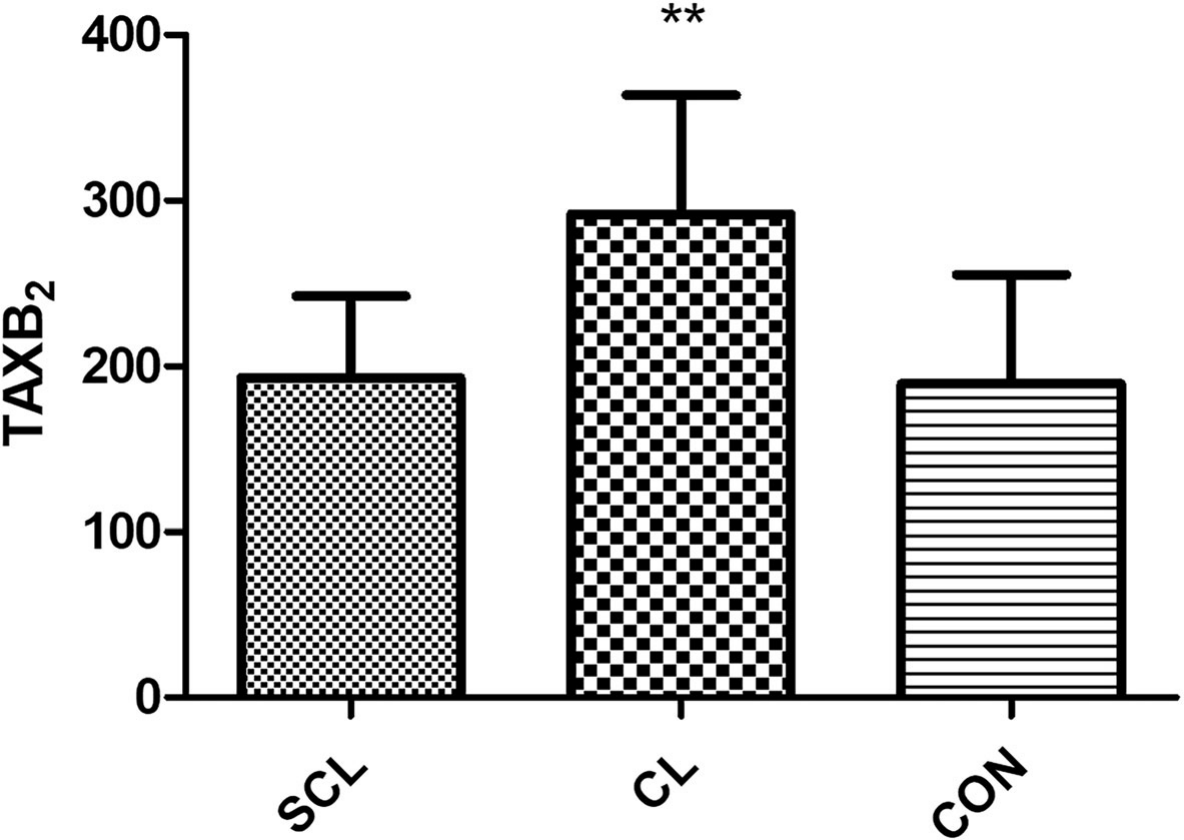
column bar plot comparing the distribution of: Thromboxan (TXB2), in 20 dairy cows with subclinical laminitis, 20 dairy cows with chronic laminitis and 20 healthy dairy cows. The error bar shows with a line, symbol * indicate statistically significant differences between groups

**Fig. 7:**
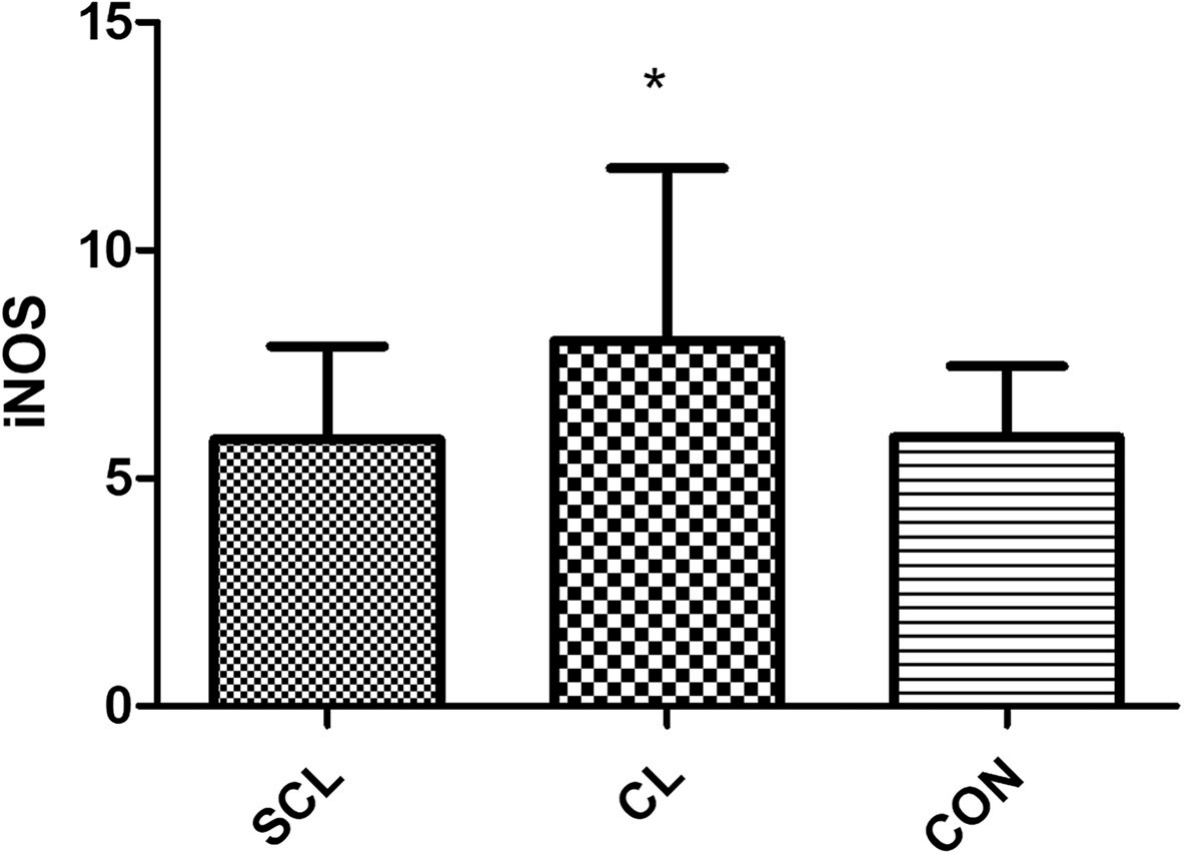
column bar plot comparing the distribution of: iNOS, in 20 dairy cows with subclinical laminitis, 20 dairy cows with chronic laminitis and 20 healthy dairy cows. The error bar shows with a line, symbol * indicate statistically significant differences between groups

From the results, compared with the healthy group, iNOS and TBX2 in the subclinical laminitis group had significant differences (*P* < 0.05), and TBX2 had extremely significant differences (*P* < 0.01) as shown in (Figs. 6, 7). Compared with the healthy group, there was no significant difference in the patients with chronic laminitis.

## Discussion

In dairy farming, mastitis, hoof and reproductive diseases are the main diseases that threaten the health of dairy cows. Laminitis is the most common form of hoof disease. Laminitis is a diffuse, serous, aseptic inflammation of the dermal papilla and vascular layers of the hoof wall [8], which can cause hoof deformation, hoof ulcer disease, white line disease as well as other hoof diseases, commencing painful anxiety in dairy cows, seriously affecting animal welfare and resulting in huge economic losses to dairy industry. Therefore, the research on the etiology, pathogenesis, diagnosis, and prevention of the dairy cow’s laminitis has important practical significance to improve the performance of dairy cows.

Because the etiology of laminitis is still controversial, its pathogenesis is inconclusive. However, the prevailing view on the pathogenesis is that when cows consume excessive amounts of concentrate, the microorganisms in the rumen, especially *Streptococcus bovis.* Adequate nutrients and suitable pH rapidly multiply into dominant strains, produce a large amount of lactic acid, endotoxin, and other vasoactive substances which enter into the systemic blood circulation to enhance the permeability of the blood vessel wall, change the blood theology index. It is an increase in the cohesiveness of red blood cells and platelets, which causes microcirculatory disorders and is prone to thrombosis [9]. On the other hand, when lactic acid accumulates in the rumen and the pH value falls below 4.5, histidine decarboxylates under the action of bacterial decomposition, producing a large amount of histamine, which acts on the hoof dermis through body fluid circulation to make capillary permeability. Additionally, along with the hoof tissue blood return resistance and long-term negative weight causing hoof tissue capillary congestion, blood stasis, blood backflow blocked, resulting in hoof local blood microcirculation disorders, hoof tissue oxygen deficiency, metabolic disorders, exudation Increased, causing cow’s laminitis [8, 10].

Studies of black walnut extract and starch models by US research institutions have shown a significant increase in the expression of inflammatory cytokines [11]. Neutrophil and platelet activation may play an important role in the development of laminitis, cytokines, interleukin-1β (IL-1β), IL-6, IL-8, cyclooxygenase-2 (COX-2), endothelial and cell adhesion factors involved in the early inflammatory response of laminitis [12]. Similar to the pathogenic model, overfeed concentrate in the pasture can lead to higher starch in the feed, which in turn causes excessive conversion of sugar in the cow’s body, leading to laminitis [13].

General bacterial toxins can be divided into two categories: exotoxin and endotoxin. Exotoxin is a toxic protein released into the bacteria while outside the bacteria during growth which is mainly produced by Gram-positive bacteria such as tetanus and diphtheria and Gram-negative bacteria. Endotoxin is also called LPS, a compound present in pathogens and bacteria is a unique structure of the cell wall of Gram-negative bacteria [14]. In general, endotoxin is different from exotoxin, and live bacteria do not secrete soluble endotoxin. It is usually released during the rapid growth and reproduction of bacteria or after death. The main pathogenic factor is LPS. Studies have shown that LPS can directly act on the mononuclear macrophages of the body, leading to the excessive release of inflammatory mediators (IL-6, platelet-activating factor, etc.), thereby inducing a chain reaction of the body [15]. In the host, it manifests as host inflammation, inhibition of immune function, disorder blood circulation, imbalance of water and salt metabolism, accumulation of metabolites, eventually leading to systemic dysfunction, and even organ failure, leading to serious death.

The study results showed that the concentration of LPS in the blood of sick cows increased significantly. This is exactly because the cows were fed refined grain, and starch decomposition leads to excessive oligofructose (OF). The small intestine cannot directly digest the OF and reacts with cecum, and the cecal bacteria digest it to reach the OF of the cecum. The normal number of bacteria cannot consume such a large amount of OF, so the intestinal bacteria multiply. Due to the large proliferation of intestinal bacteria, the intestinal pH decreases, the intestinal environment is not suitable for bacterial survival and proliferation, which in turn causes the death of Gram-negative bacteria, resulted in increased LPS content in the intestine. Decreased pH and excessive LPS can damage the intestinal mucosa, resulting in moderate-to-severe enterocolitis [16]. Intestinal mucosal barrier damage causes a variety of substances, including LPS, to be absorbed into the blood, causing systemic inflammatory reactions with diarrhea.

Tumor necrosis factor-α (TNF-α), a pro-inflammatory cytokine that plays a key role in metabolic syndrome and pathological processes. TNF-α is a key mediator of acute and chronic inflammatory responses. It plays an important role in the development of autoimmune and tumor diseases [17, 18]. It is mainly produced by activated macrophages, including macrophages located in adipose tissue [19], but TNF-α is also produced by various other cells, such as lymphoid cells, mast cells, endothelial cells, cardiac muscle cells, Adipocytes, fibroblasts and neurons [20].TNF-α has a wide range of biological activities and has a strong anti-tumor effect. It is the most potent cytokine discovered so far [21]. In addition to direct inhibition of proliferation and necrosis of tumor cells, it also affects other cells including the growth and differentiation of cardiomyocytes also have an effect, as well as anti-virus and bacteria, activate T cells, promoted the production and secretion of IL-1, IL-2, and IL-6, induce inflammatory reactions, and promote IL-2R and epidermal growth factor receptor (EGFR). At low concentrations (10^− 10^ mol/L), TNF-α acts as an autocrine and paracrine regulator of leukocytes and endothelial cells and is involved in the fight against bacteria, viruses and parasites infection, promote tissue repair and regulate the inflammatory response, cause tumor cell apoptosis, etc. At high concentrations (≥10^− 8^ mol/L), excessive production and release of TNF-α in the body destroy the body’s immunity balance, along with other inflammatory factors, produce a variety of pathological damage. TNF-α has biological effects on many tissues and organs, suggesting that it may be an important multi-functional member of the cytokine network. It is an essential immune regulator for the body to maintain internal self-stability and resist various pathogenic factors. In this experiment, the TNF-α of the affected cows in the subclinical laminitis group was significantly higher than that of the healthy group (*P* < 0.05), indicating that TNF-α was involved in the occurrence of laminitis.

Histamine is a vasoactive molecule, when exposed to high concentrations, causes the body to collapse and promotes the secretion of pepsin and gastric acid. When ruminant acidosis occurs, the content of histamine in the rumen fluid and blood increased, reaching the hoof through the blood circulation, causing the capillary permeability of the local microcirculation to become larger and the expansion of the small artery which is related to the laminitis [22]. When the hoof tissue is affected, damage immune response, allergen, surfactant, and other factors, the histidine stored in the mast cell is decarboxylated and converted into histamine, which is released into the blood circulation, thus affecting the hoof microcirculation [22].

COX-2 is mainly expressed in glomerular dense plaques, cortical medullary ascending branches, podocytes and interstitial cells of the renal medulla. Besides, the expression of immune-reactive COX-2 in glomerular visceral epithelial cells and mesangial cells was also increased after subtotal nephrectomy. COX-2 and inflammatory mediators were involved in glomerular and tubular interstitial structural changes. Hemodynamic abnormalities were the earliest manifestations of diabetic nephropathy. Studies have shown that the high expression of COX-2 in rat vascular smooth muscle cells increases the responsiveness of vasoconstriction, which may affect renal hemodynamics [23]. On the other hand, COX-2 metabolites PGE1 and PGI2 can dilate renal blood vessels and inhibit platelet aggregation, thereby increasing renal blood flow and glomerular filtration rate; while thromboxane A2 (TXA2) acts just the opposite.

NO is a cell-transporting substance that is important in vascular endothelial cells. It is thought to be a messenger molecule and an effector molecule that performs functions in the body. It catalyzes the production of cyclic guanosine monophosphate, expands blood vessels, and increases permeability. It is beneficial for inflammatory mediators and pain-causing substances to reach the site of action, increasing the infiltration of monocytes into the inflammatory site in the inflammatory response. In the inflammatory response, inducible nitric oxide synthase (iNOS) was activated, thereby synthesizing excess NO, which was toxic to cells, triggering immunopathological processes and causing tissue damage and aggravating inflammatory response [24]. In our study, the iNOS of cows in the chronic laminitis group was significantly higher than that in the healthy group (P<0.05). Because NO was catalyzed by inducible nitric oxide synthase (iNOS) to catalyze the production of L-arginine and molecular oxygen in the inflammatory response. The number of iNOS and the level of activity directly determine the amount of NO produced. The basic amount of NO has the effect of preventing vasospasm and thrombosis, but under conditions such as trauma and sepsis, the level of NO in the body increases exponentially, and high concentration of NO produces an oxidation reaction that damages the body [25].

Interleukins are the groups of cytokines produced by a variety of cells that play an intermediate role in intercellular signaling and affect the immune system. Some rare interleukin defects usually lead to autoimmune diseases or immunity defects. IL-6 is a cytokine of interleukins, mainly composed of antigen-presenting cells (macrophages, dendritic cells), B cells, T cells, non-hematopoietic cells such as epithelial cells, endothelial cells, astrocytes, fibroblasts, osteoblasts, mesangial cells and tumor cells [26], causing acute phase reactions of inflammation. In our experiment, the levels of IL-6 in the diseased groups were significantly increased (*P* < 0.05), which was positively correlated with the LPS content of the diseased group. LPS can stimulate the production of IL-6. IL-6 itself has no direct damage to cells, but IL-6 can promote the activation and aggregation of neutrophils and cause damage to multiple organ tissues [27]. IL-6 is a potent inhibitor of adrenergic vascular smooth muscle contractile material. Therefore, an increase in plasma IL-6 levels not only increases the concentration of NO but also dilates the blood vessels by reducing the reactivity of the vascular endothelium to the vasoconstrictor.

The above results showed that in the subclinical laminitis group, the comparison between the two groups in the chronic laminitis group and the healthy group were not completely different. This may be due to subclinical laminitis. The cause of the disease is different from the cause of chronic laminitis.

As a result, the horny horn of dairy cows suffering from subclinical laminitis become soft within a few weeks. We found in the dairy cattle farm that the floor of the barn was uneven, which made the hoof unevenly affected and damaged the horny horniness, causing the hoof congestion and laminitis. When a sick cow walks on hard and uneven ground, the soft horny skin becomes wear out; the bottom of the hoof is damaged and shows bleeding at the bottom of the hoof [28].

The results showed that there were many types of cow hysomitis in the pasture. We had analyzed the blood of the most common type of laminitis type cows and related inflammatory factors and vasoactive substances. The undulation of the ground was closely associated with laminitis.

## Conclusion

This study provides a first report of that The pasture laminitis was subdivided into subclinical laminitis and clinical chronic laminitis, and the results were different from other studies. In our findings, we found that not all indicators of laminitis have similar results. It is revealed that chronic laminitis caused to abnormal metabolism in cattle, while subclinical laminitis is more due to the external environment. Through the study of inflammatory factors and vasoactive substances, it is found that they have a close relationship, which affects the metabolic cycle, abnormal expression of inflammatory factors and vasoactive substances in dairy cows, and causes hoof microcirculation disorders. The interaction is accompanied by the development of laminitis, which provides a theoretical basis for an in-depth understanding of the mechanism of development and development of cow’s laminitis.

## Methods

### Farm and animal selection

**Fig. 8:**
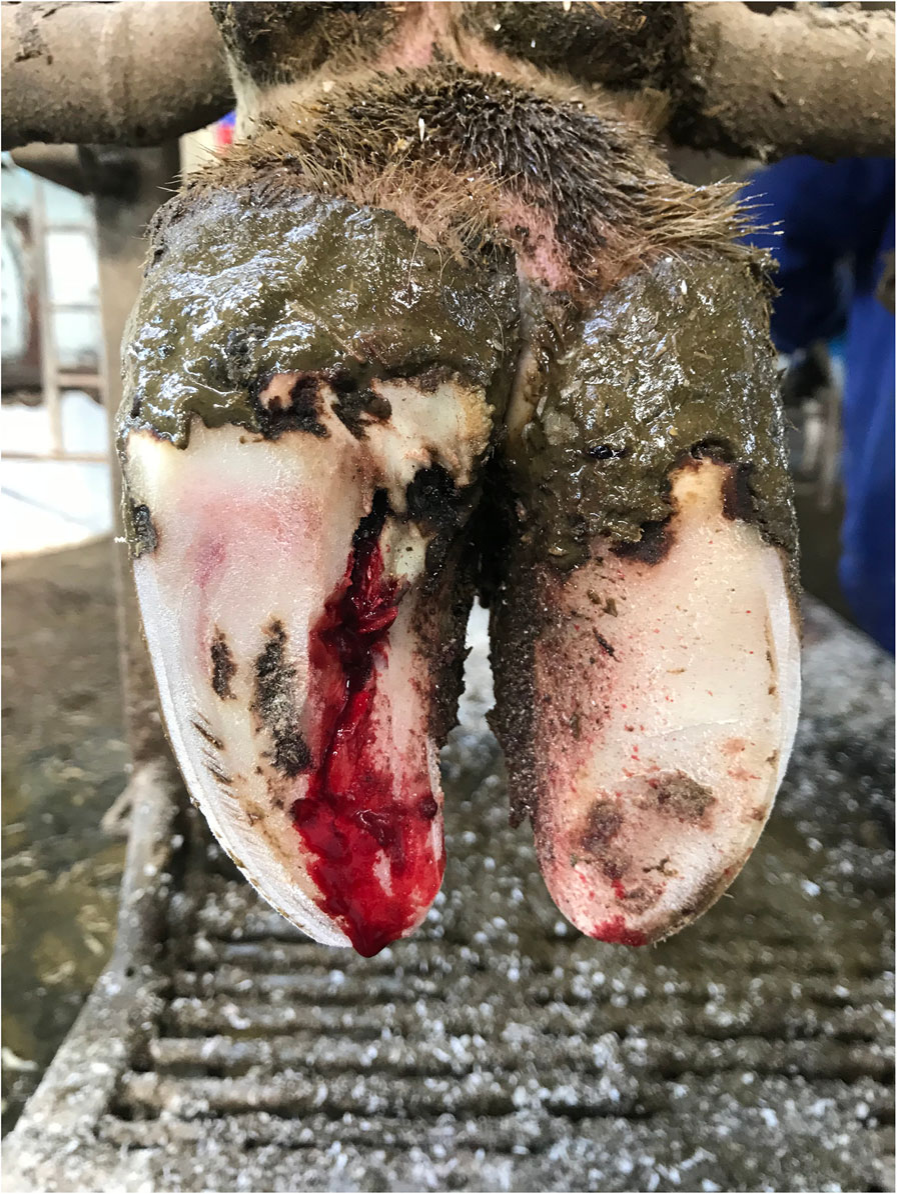
The hoof of subclinical laminitis cows

**Fig. 9:**
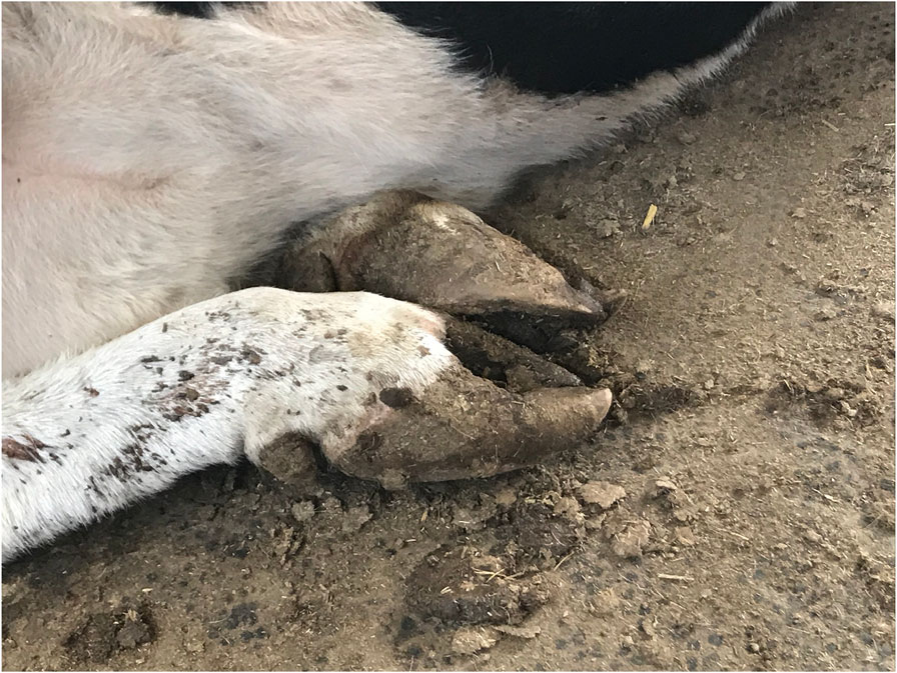
The hoof of chronic laminitis cows

**Fig. 10:**
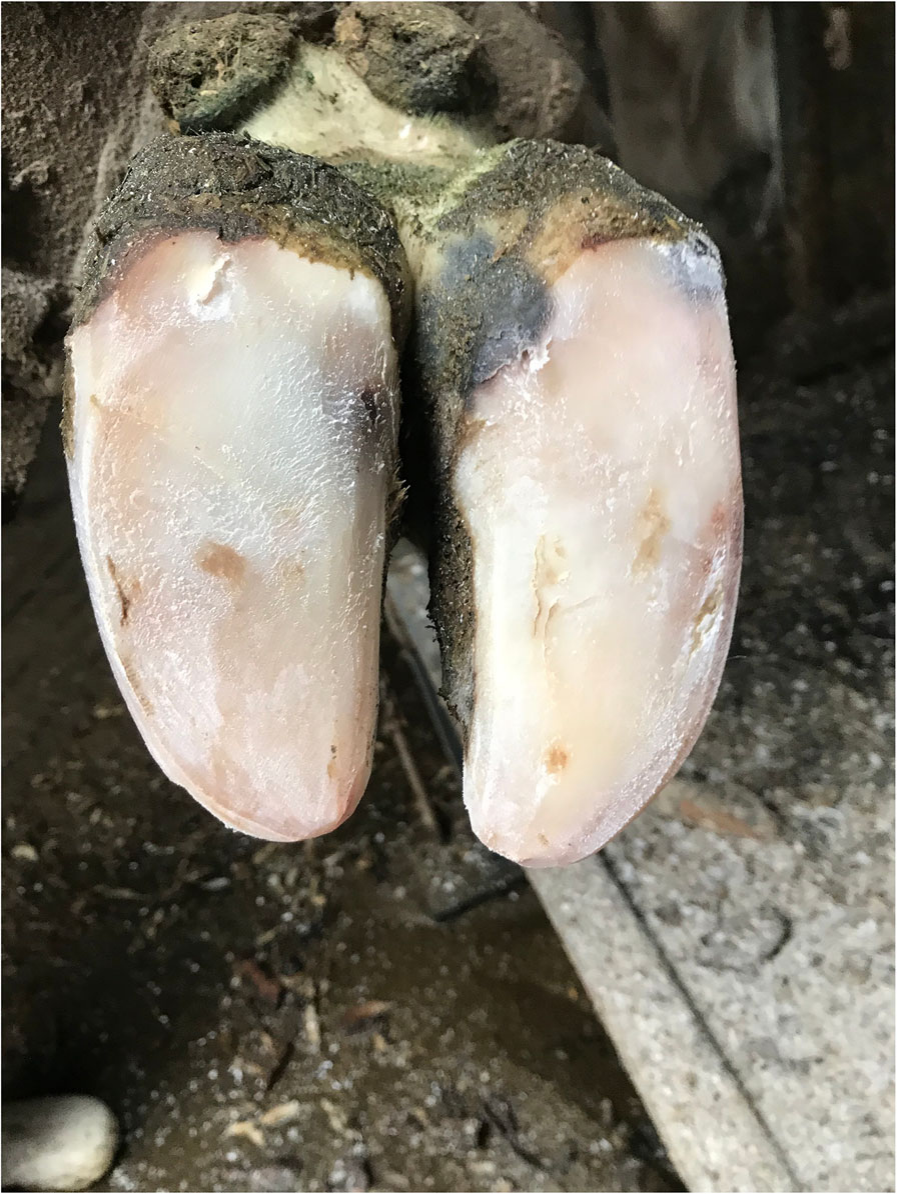
The hoof of healthy cows

The cows with (SCL) subclinical laminitis (*n* = 20) (Fig. 8) and (CL) chronic laminitis (*n* = 20) (Fig. 9) were selected from a local dairy farm in Harbin City, Heilongjiang Province, China. All the dairy cows with subclinical laminitis and chronic laminitis were diagnosed by pasture veterinarians. Diagnostic standards: The lesion characteristics of subclinical laminitis are mainly hoofed lesions, such as hemorrhage in the hoof and white line, as well as a sole ulcer [1]. The pathological features of chronic laminitis are hoof deformation, ball weight and bottom weight of hoof are not accurate, and the hoof is extended [28]. Besides, 20 cows (CON) (Fig. 10) without any disease were randomly selected as a healthy control group. The cattle farm was routinely raised and managed. The experimental diet was fed based on the daily feed formula at dairy farms. All of the three groups of cows were fed with the same basic diet and drinking water.

### Sampling

All dairy cows were gathered from the pasture at the same time of day (between 08:00 am to 12:00 pm) to collect blood samples through venipuncture for seven consecutive days in early October 2018. All the collected blood samples were transferred into heparinized and EDTA vacutainer tubes as well as 0 °C refrigerated until centrifugation. Plasma was removed from blood within 30 min and stored at − 80 °C until analyzed for vasoactive substances and inflammatory mediators (Histamine (HIS), lipopolysaccharide (LPS), tumor necrosis factor-α (TNF-α), interleukin-1β (IL-1β), interleukin 6 (IL-6), cyclooxygenase-2 (COX-2), 5-hydroxytryptamine (5-HT), thromboxane-2 (TBX-2), endothelin-1 (ET-1), inducible nitric oxide synthase (iNOS), and prostacyclin-2 (PGI-2)). The ELISA kits (Shanghai Meilian Bioengineering Institute, Shanghai, China) were used to measure all the study parameters. The assay for all the parameters was performed according to the manufacturer’s instructions.

### Experimental procedure

The desired slats were removed from the foil pouch after equilibration for 60 min at room temperature. Standard wells, blank wells and sample wells were set, and standard wells were each added with 50 μl of different concentrations of standard. Add 50 μl of the sample to be tested to the sample well; add 50 μl of the sample dilution to the blank well. 100 μl of horseradish peroxidase (HRP)-labeled detection antibody was added to each well of blank well, standard well and sample well, and the reaction well was sealed with a sealing membrane, and incubated for 60 min in a 37 °C water bath or incubator. Discard the liquid, pat dry on the absorbent paper, add the washing solution (350 μl) to each well, let stand for 1 min, remove the washing solution, pat dry on the absorbent paper, and repeat the washing 5 times. 50 μL of each of the substrates A and B was added to each well, and incubated at 37 °C for 15 min in the dark. 50 μL of the stop solution was added to each well, and the OD value of each well was measured at a wavelength of 450 nm within 15 min. The standard product concentration is plotted on the abscissa and the OD value is plotted on the ordinate. The linear regression curve of the standard is drawn, and the concentration values of each sample are calculated according to the curve equation.

### Statistics analysis

All statistical analyses were performed using SPSS 22 version for Windows. The normality of data was assessed using the Kolmogorov-Smirnov test. The indicator for each cow was analyzed using the one-way variance of Dennett’s post hoc analysis. The results were expressed as (mean ± SD) and considered significant (*P* < 0.05; *P* < 0.01).

## Data Availability

The datasets used and/or analysed during the current study are available from the corresponding author on reasonable request.

## Abbreviations

SCL: Subclinical laminitis
CL: Chronic laminitis
5-HT: 5-Hydroxytryptamine
ET-1: Endothelin-1
PGI-2: Epoprostenol
COX-2: Cyclooxygenase-2
HIS: Histamine
IL-1β: Interleukin-1β
IL-6: Interleukin-6
LPS: Lipopolysaccharide
TNF-α: Tumor necrosis factor-α
TXB2: Thromboxan
iNOS: Inducible nitric oxide synthase
